# Unraveling Mental Health Stigma: A Qualitative Study of Health Science Students' Perceptions Toward Patients With Mental Illness in the United Arab Emirates

**DOI:** 10.1111/phn.70007

**Published:** 2025-08-05

**Authors:** Jacqueline Maria Dias, Mariam Rashid Alhmoudi, Halima Abdalla Aldhuhoori, Amira Mohamed Raeisi, Noura Saeed Alnaqbi, Omar Ahmed Najjar, Fatma Refaat Ahmed, Mini Sara Abraham, Richard Mottershead, Nabeel Al Yateem, Ambrose Richard Dias, Muhammad Arsyad Subu

**Affiliations:** ^1^ Department of Nursing, College of Health Sciences University of Sharjah Sharjah UAE; ^2^ University of Wollongong Dubai UAE

**Keywords:** baccalaureate students, campus, health science, mental illness, qualitative methodology, stigma

## Abstract

**Background:**

The stigma surrounding mental health is a key public health issue that can impede timely help‐seeking and weaken the therapeutic relationships between individuals with mental health conditions and healthcare professionals. Although stigma among health profession students in the Arab region has been studied, its exploration among United Arab Emirates health science students remains limited. This gap highlights the novelty and significance of the present study.

**Objective:**

This study explored attitudes related to mental health stigma toward individuals with mental illnesses among Year 3 baccalaureate students.

**Design:**

A qualitative, exploratory, and descriptive approach was used.

**Methods:**

Semi‐structured interviews were conducted with 25 students enrolled in seven healthcare programs. Thematic analysis was adopted for the data analysis.

**Results:**

Four distinct interrelated themes were extracted from our content analysis: (1) perception and knowledge of mental health, (2) unpacking stigma surrounding mental illness, (3) attitudes toward mental health, and (4) advocacy and education: recognizing the urgency of promoting mental health awareness and initiatives.

**Conclusions:**

Students generally held positive views on mental health, though some expressed stigma and noted insufficient education depth. These findings suggest the need for targeted curriculum interventions to better prepare health science students for future roles.

## 1 Relevance of This Paper

This study explores the attitudes of Year 3 health sciences students toward individuals with mental illnesses across disciplines, including nursing, physiotherapy, medical laboratory sciences, nutrition and dietetics, medical diagnostic imaging, environmental health, and healthcare management, in the United Arab Emirates. Although previous studies have examined stigma among health science students, limited research exists in the Arab context, making this study particularly meaningful. Targeted interventions and supportive mechanisms can be built into the curriculum to support health science students’ preparation for future roles.

### 1.1 Background

Mental health encompasses individuals’ ability to engage in productive daily activities, maintain healthy relationships, and effectively manage challenges and changes (Koppenborg et al. 2024). It is a critical element of emotional and personal well‐being, influencing one's thoughts, emotions, actions, interactions with others, stress management, and health‐related decisions (World Health Organization [WHO] 2022). Mental health is integral to holistic well‐being and includes life's social, emotional, and psychological aspects.

Mental health problems, also referred to as mental illnesses, represent a wide range of conditions distinguished by considerable distress, impaired functioning, and the risk of self‐harm (WHO 2022). Despite the medical nature of mental health issues, many individuals avoid seeking treatment because of concerns about their family's reputation, diminished marriage prospects, societal exclusion, and stigma (WHO 2019).

Individuals with mental health problems often experience lower self‐esteem, academic struggles (Bruffaerts et al. 2018), and strained relationships with their families and peers (Lien et al. 2021). Moreover, they are at a higher risk of developing physical health conditions such as cardiovascular diseases, respiratory illnesses, and diabetes (Correll et al. 2017). Mental illness is a treatable medical condition, like diabetes or cardiovascular disease, and should not cause shame (Correll et al. 2017).

Stigma is an attribute that is deeply discredited; it reduces a person's social status and devalues their identity (Goffman 1963, 3). Building on Goffman's work, Corrigan conceptualizes stigma as a multifaceted process comprising public stigma, self‐stigma, and label avoidance (Corrigan et al. 2001). Furthermore, Corrigan (2004) found that stigmatizing experiences could exacerbate feelings of exclusion and inadequacy, hindering individuals from seeking or continuing treatment (Corrigan 2004). It is estimated that stigma affects four out of every five individuals with mental illness (Mohammadzadeh et al. 2020), leading to adverse outcomes such as reduced self‐esteem, family conflicts, and limited employment opportunities (Picco et al. 2019; Subu et al. 2021). Stigma affects individuals on a societal level and is deeply embedded within the healthcare system, where it manifests across structural, interpersonal, and intrapersonal domains. A person with a mental illness might face social rejection, isolation, and discrimination throughout much of their life (Corrigan and Bink 2015). Mentally ill individuals frequently articulate sentiments of dehumanization, rejection, and devaluation during their interactions with healthcare practitioners. Despite their training, healthcare professionals are not immune to stigmatizing attitudes, which can significantly affect the treatment and care of individuals with mental illnesses (Subu et al. 2021). They are more likely not to adhere to or follow medical treatments for other health issues. The bias and discrimination associated with the stigma of mental illness significantly contribute to ineffective treatments and reluctance to seek care (Corrigan et al. 2014).

Historically, healthcare professionals have been believed to be immune to the challenges faced by patients with mental health conditions (Chow et al. 2020). However, evidence suggests that stigmatizing attitudes are prevalent even among health professionals, including medical and nursing students, with many exhibiting negative stereotypes and pessimistic expectations of recovery. For instance, studies show that psychiatric students may view people with mental illness as dangerous and unpredictable (Desai and Chavda 2018). Despite this, there is limited understanding of how students perceive their comfort in diagnosing or treating mental illnesses, their attitudes toward disclosure, and their perceptions of the mental health field (Masedo et al. 2021). Studies suggest that educational interventions can significantly diminish stigma, particularly among health care professionals lacking formal mental health training (Corrigan 2004, Henderson et al. 2014). Subu et al. (2024) found that Indonesian students generally hold positive attitudes toward individuals with mental illness, with many reporting that such encounters enhanced their understanding of the associated challenges.

People facing mental health challenges often endure feelings of dehumanization, rejection, and devaluation when interacting with healthcare providers (Knaak et al. 2017). This stigma can notably hinder their access to therapy. Some individuals may be reluctant to seek help due to fears of societal stigma or discrimination associated with mental health issues. Social constructionism posits that our cultural interactions significantly shape our reality, highlighting the importance of a shared history of values and customs in advancing mental health (Corrigan and Rusch 2002). A lack of awareness regarding mental illnesses often hampers access to professional mental health services (Corrigan and Bink 2015; Subu et al. 2022). Sociocultural perspectives on mental illness can strongly affect individuals' decisions about service use, treatment options, and their responses to professional mental healthcare (Corrigan 2004).

Stigma surrounding mental illness in the United Arab Emirates is deeply shaped by cultural, social, and religious influences, often leading individuals and families to reject formal mental health services. Some perceive mental health clinics as inadequate, believing practitioners neglect religious values, prompting reliance on traditional healers instead (Bhikha et al. 2015). Misconceptions such as attributing mental illness to evil spirits, black magic, or the evil eye are widespread in some Arab communities (Alahmed et al. 2018; Subu et al. 2022). These beliefs contribute to significant stigma, fostering feelings of shame and reluctance to discuss mental health openly, as mental illness is sometimes perceived as a personal or familial weakness (Fekih‐Romdhane et al. 2023). Consequently, families may discourage help‐seeking and instead turn to religious figures, further delaying appropriate treatment (Fekih‐Romdhane et al. 2023; Merhej 2019; Subu et al. 2023).

At the University of Sharjah, the College of Health Sciences follows a competency‐based undergraduate curriculum across all seven departments: Medical Laboratory Sciences, Nursing, Physiotherapy, Medical Diagnostic Imaging, Healthcare Management, Nutrition and Dietetics, and Environmental Health Sciences. This approach emphasizes the development of professional competencies, including communication, ethical practice, and psychosocial understanding, as well as skills essential to providing holistic care. Understanding the curriculum context is important when exploring health science students’ perceptions and attitudes, particularly regarding mental health, as it shapes their academic and clinical exposure to stigma.

Despite emphasis on mental health in the curriculum, there are key gaps in the education provided to students, particularly concerning mental health stigma. Students receive only a single three‐hour session on stigma in their second year under the “Psychosocial Aspects in Healthcare” course. Crucial areas such as comfort in managing mental illness, attitudes toward disclosure, and perceptions of the mental health field are insufficiently addressed in the curriculum.

This study explored attitudes related to mental health stigma toward individuals with mental illnesses among Year 3 students at the College of Health Sciences. Although previous studies have examined the stigma toward mental illness among health science students, there is limited research on this issue in the Arab region. This gap in the literature is the novelty and significance of this study, as it seeks to provide insights into how cultural factors may shape stigma and pave the way for curriculum reform that equips students with the knowledge and skills needed to reduce stigma and support individuals with mental illnesses effectively.

## 2 Methods

This study explored mental illness stigma among healthcare students toward individuals with mental illnesses in the UAE using a descriptive qualitative approach. The knowledge, attitudes, and behavioral responses of healthcare students with mental illnesses were analyzed and identified using thematic analysis to reveal patterns, meanings, and themes (Braun and Clarke 2006). Qualitative research collects and analyzes data gathered through interviews and observations. Qualitative research explores the meanings of social phenomena as perceived by people in particular circumstances, focusing on how people understand their experiences and the meanings they give to them (Grossoehme 2014).

### 2.1 Settings and Participants

This research took place in the Emirate of Sharjah, United Arab Emirates. To ensure maximum variability, purposive sampling was employed to select participants from all seven healthcare programs: nursing, physiotherapy, medical lab sciences, nutrition and dietetics, medical diagnostic imaging, environmental health, and healthcare management at the College of Health Sciences. In purposive sampling, individuals are intentionally selected based on specific characteristics or criteria pertinent to the research question. The goal of purposive sampling is not to represent the entire population but to collect detailed participant data. This ensures a diverse range of perspectives and experiences of mental health stigma. The sample was balanced for gender: 12 male and 13 female students. This study focused on Year 3 students, who had completed foundational coursework and had some clinical exposure, to capture their attitudes and perceptions of mental illness and stigma at a critical stage in their education.

### 2.2 Research Instrument

An extensive review of relevant literature and previous studies on mental health stigma was conducted to formulate the interview questions. It included keywords in Google Scholar and PubMed with the terms “health sciences students,” “stigma,” “mental illnesses,” and “mental health.” These interviews aimed to provide insights into the attitudes and perspectives of healthcare students toward mental illness. Once the questions were created, they were validated by two other faculty members with relevant mental health experience. A pilot test was conducted with five students, and based on their feedback, the following research questions were addressed:

1. Can you share any personal encounters or interactions you have had with individuals experiencing mental health challenges?
2. From your perspective, how would you describe your understanding of mental illnesses?
3. How do you perceive your level of empathy and compassion toward individuals living with mental health conditions?
4. If you were to face a mental health issue, could you elaborate on the steps you might take or the support you would seek?
5. What experiences, if any, have you had in professional or personal settings that involved engaging with individuals coping with mental health issues?
6. Regarding your academic training, what kind of information or insights have you gained about mental illness and its impacts?

### 2.3 Data Collection

Semi‐structured interviews were used as the primary data collection method. Interviews with participants provided an understanding of health science students’ attitudes and perspectives regarding stigma and mental illness, and a predetermined set of questions guided the semi‐structured interviews. The recruited students participated in interviews between April and May 2023. The researchers contacted each student who indicated interest in participating via email. Before each interview, participants were briefed about the research activity and asked to read and sign a consent form. The interviews were conducted in a classroom, away from distractions, and typically lasted between 30 and 40 min. All interviews were conducted by J.M.D. and M.A.S., who have backgrounds in mental health. During the interviews, participants were encouraged to share their experiences and viewpoints on various aspects of mental health stigma. To ensure that participants felt comfortable and not under pressure, they were given ample time to reflect and articulate their responses. Data collection continued until rich, in‐depth accounts were obtained across participants, and no new themes emerged, suggesting that sufficient depth and diversity of perspectives had been captured and data saturation was reached.

### 2.4 Data Analysis

This study employed reflexive thematic analysis, following Braun and Clarke's (2006) six‐phase framework to guide the analytical process (Braun and Clarke 2006). In the initial phase, one team member carefully checked the verbatim interview transcripts for accuracy and familiarized themselves with the data through active reading. The data were then systematically coded to capture units of meaning relevant to participants' lived experiences and perceptions of mental health. Coding was conducted inductively, comparing their similarities and differences in codes throughout the research to explore patterns of meaning across the dataset. The first author constructed preliminary themes, which were refined in discussion with the broader research team to enhance depth and clarity. These themes were then reviewed in relation to the entire dataset and relevant literature to ensure they meaningfully captured students’ contextual experiences.

### 2.5 Study Rigor

After extracting relevant excerpts from participants' accounts, we compared the codes and units of meaning to identify similarities and differences. Based on this process, abstract themes were developed to elucidate experiences in line with existing literature. We followed the qualitative data rigor standards proposed by Chiovitti and Piran (2003) to ensure rigor. This included adherence to principles of credibility, auditability, and transferability. Credibility was maintained through accurate representations of participants’ experiences, achieved through meticulous manual classification and analysis. Auditability was ensured by making our methodologies and findings accessible to other researchers, allowing for verification and replication. Transferability was demonstrated by establishing the relevance of our findings to similar contexts; clarifying the relationship between our findings and existing literature; and defining the scope of our research regarding participants, location, and thematic intensity.

### 2.6 Ethical Considerations

This study was performed following the procedures outlined in the Declaration of Helsinki. This study was approved by an appropriate research and ethics committee. After obtaining ethical approval, the researchers approached potential participants in each program and explained the study's relevance, methodologies, and objectives. Potential participants were informed that their participation was at their own discretion, that they had the right to withdraw from the study at any time, and that their decision to engage or abstain would not have any impact on their subsequent studies or grades. Written informed consent was obtained from participants before data collection, and measures were taken to maintain the confidentiality and anonymity of their information. To ensure that the transcriptions were not related to participants, everyone was assigned a distinct alpha‐numeric designation (e.g., P1 represented participant 1, P2 represented participant 2, and so on). Each interview was uploaded to a password‐protected computer. The entire interview material, encompassing transcriptions and recordings, was accessible only to the researchers.

## 3 Findings

### 3.1 Participants’ Demographics

Twenty‐five health science students enrolled in their respective programs' third year participated (Table 1). Following the content analysis, we identified four main themes with sub themes, which were distinct yet interconnected: (1) perception of knowledge of mental health with two sub themes: (a) feelings of empathy with people having mental illness and (b) familiarity and knowledge about mental illness; (2) unpacking stigma around mental health with five sub themes (a) public stigma, (b) indifference and rejection, (c) sources of stigma surrounding mental illness, (d) consequences of stigmatizing mental illness, and (e) mental illness and violent behavior; (3) attitudes toward mental illness; and (4) advocacy and education: recognizing the urgency of promoting mental health awareness and initiatives. An account of each theme is presented, along with supporting excerpts for the identified themes. The identified themes provided valuable insights into understanding stigma and mental health from the perspective of health science students. These themes will lead to targeted interventions and supportive mechanisms that can be built into the curriculum to support health science students in preparing them for their future roles as health professionals.

**TABLE 1:** Demographic data.

**Sex**		
Female	13	52%
Male	12	48%
**Age (years)**		
18–22	22	88%
23–27	3	12%
**Ethnicity**		
UAE nationals	3	12%
GCC countries	15	60%
Africa	5	20%
Southeast Asia	2	8%
**Year of study**		
Year 3	25	100%

### 3.2 Theme 1: Perception and Knowledge of Mental Health

This theme revolved around exploring the understanding and misconceptions about mental health. This theme was further subdivided into two themes: feelings of empathy with people with mental illness and familiarity and knowledge about mental illness.

#### 3.2.1 Feeling of Empathy With People With Mental Illness

Some participants acknowledged their personal encounters and familial connections with mentally ill individuals. Additionally, some participants shared previous clinical encounters in caring for patients with mental illness. A student said:

“You know…. I personally have dealt with mental health issues. I am able to offer insights and knowledge regarding the system for mental health care, but I strongly believe my knowledge was limited. Like my family and close acquaintances, I have been exposed to individuals with a wide range of mental illness.” (Participant 19).

Participants believed that mental illnesses did not have a meaningful presence in daily life and advocated for equal treatment of those with mental and physical illnesses. A student said:

“…I am convinced that everyone will also possess these [problems]…It is our duty to ensure that the patient has a secure environment, regardless of the fact that they [patients] are living with mental illness. Moreover, they ought to be regarded as ordinary individuals.” (Participant 5).

#### 3.2.2 Familiarity and Knowledge About Mental Illnesses

Participants showed a basic understanding of mental illnesses, recognizing causes such as genetic predisposition and social/environmental, and cultural factors. However, many respondents expressed a lack of familiarity with managing patients with severe mental illnesses. One student said:

“While I am equipped with the means to recognise certain issues and consider them potentially problematic, I am far from being sufficiently educated to offer substantial assistance to others with mental illness.” (Participant 15).

Another student stated:

“It is not culturally acceptable to talk about mental illness and stigma in my family, and I have seen my family members delay the possibility of obtaining treatment.” (Participant 13).

### 3.3 Theme 2: Unpacking Stigma Surrounding Mental Illness

This theme revolves around stigma dynamics and the unpacking of the social constructs of mental illness. This theme was further broken down into five subthemes: public stigma, indifference and rejection, sources of stigma surrounding mental illness, consequences of stigmatizing mental illness, and violent behavior.

#### 3.3.1 Public Stigma

Most students recognized the societal stigma surrounding mental illness and noted its persistent negative perceptions. Most students recognized the societal stigma associated with mental illnesses. This negative societal perception of mental illness is reflected in the following statement:

“It represents a societal disgrace and stigma. Many individuals did perceive those who suffered from mental illnesses as unstable, unpredictable, disoriented, and dangerous. This is a fact within our society. Although I maintain the view that a stigma…It appears to be diminishing, or so I hope.” (Participant 4).

Another participant reiterated a similar statement in the following words.

“Stigma is diminishing in our society, in my opinion, but will take time; people need to be educated about stigma.” (Participant 12).

#### 3.3.2 Indifference and Rejection

Students’ lack of consistent attention, avoidance, or exclusion of individuals with mental illness constituted a negative attitude. Although uncommon, this perspective appeared among students of both sexes, primarily indicating a reluctance to work with or interact with an individual experiencing a mental illness. A student said:

“While unfair prejudice is unacceptable, discrimination itself is not wrong, right? For instance, if I were a manager scheduling an interview with a worker and discovered that he suffers from a mental illness, I think, for me, I would not appoint him to the position, as it would be detrimental to the mental health of the customers. First, he should receive appropriate treatment for his condition.” (Participant 12).

The participant in the interview used the term “rejection” to mean that he believed it is preferable to refer the patient to another professional for a range of reasons, including a lack of expertise and the inability to manage a patient with this type of illness. A student said:

“Therefore, in situations where we are unqualified or unwilling to address patients with mental illnesses, we would rather refer them to another professional and delegate the problem to that person.” (Participant 25).

#### 3.3.3 Sources of Stigma Surrounding Mental Illness

Students acknowledged the profound impact of stigma on various domains of life. Patients struggle to cope with daily challenges, which leads to hesitation in revealing their condition or seeking help. Mental illness often evokes feelings of alienation, disconnection, and marginalization. A student said:

“…It started from my own perception that he/she was inadequate. Also, I believe ignorance regarding mental illness is the source of stigma. In other words, one that promotes awareness of mental illness without advocating for those with mental illness was deemed to exacerbate the stigma associated with mental illness.” (Participant 18).

#### 3.3.4 Consequences of Stigmatizing Mental Illness

Students recognized the impact of stigmatization on various aspects of life. Patients were perceived as incapable of coping with daily challenges, leading to a reluctance to disclose their condition. According to students, patients were regarded as inadequate and ill‐equipped to confront difficulties in their daily lives. There was a common tendency among patients with mental disorders to be reluctant to disclose their conditions. Mental illness creates feelings of alienation, disconnection, separation, and marginalization. A student said:

“A social stigma pertains to a society where people are judged on the basis of their capabilities…It would appear that they are attempting to conceal the fact that they have a mental illness. The absence of substantial interpersonal connections between mental ill persons and those who do not hinders the capacity to accurately perceive reality and leads to a dearth of understanding. The resultant anxiety and confusion are exhibited as stigma.” (Participant 4).

Another student commented,

“It is best not to let anyone know you have a mental illness, for the stigma will attach to the person forever, and culturally it is not acceptable; this will affect obtaining a marriage proposal.” (Participant 22).

#### 3.3.5 Mental Illness and Violent Behavior

Participants predominantly expressed fear, particularly concerning the potential for harm or unstable behaviors associated with mental illness. This attitude is primarily referenced to potential harm or unstable behavior. A student said:

“People are fearful and me too. Yes, they [patients] must be hospitalised. Their fear prevents them from engaging in interactions with them [patients] normally. Then, it is better for their health and the individuals whose presence may influence their behaviour that they be hospitalized. It is possible that I do not know precisely what will happen…Someone must treat them with care.” (Participant 19).

### 3.4 Theme 3: Attitudes Toward Mental Illness

This theme reflects perspectives on the attitudes of mental illness of healthcare students toward individuals with mental illness, emphasizing the importance of empathy and respect within social interactions. Participants highlighted the need for fairness and consideration, advocating for equal treatment and the inclusion of individuals with mental illnesses in everyday life.

This perspective is understood when healthcare students embrace and respect the beliefs of individuals with mental illnesses in a social setting. This primarily pertains to issues of fairness and consideration. According to participants, mental illnesses are not phenomena that should be excluded from daily life, and these individuals should receive equal treatment. A student said:

“I believe we all have this feeling that we are obligated to provide a secure environment for the patient; although they have a mental illness, it is our responsibility as healthcare professionals. Furthermore, we must regard them as normal individuals, as their mental illness does not define them as individuals. Mental illness is extremely prevalent; anyone on your side could have one. Because of this, you should modify your perspective, consider an alternative treatment, and so forth.” (Participant 23).

Another student commented:

“If it becomes known that you or a colleague have a mental illness, and you are stigmatised it will affect your academic performance; so, best to be quiet and not make it known to anyone in the university.” (Participant 18).

### 3.5 Theme 4: Advocacy and Education: Recognizing the Urgency of Promoting Mental Health Awareness and Initiatives

This theme advocates the critical need for mental health awareness and initiatives. Health science students were vocal and openly advocating for increasing the focus on mental health, integrating instructional components on mental illness at an earlier stage in their respective curricula, expanding student interactions with clients who have mental illnesses, and implementing campaigns to reduce the social stigma associated with mental illness. To combat the stigma associated with mental illness, it is vital that students are informed about mental health education and awareness. One student stated.

“Indeed, that is true…The most crucial aspect is that it could be advantageous for students to interact with individuals who are experiencing mental health challenges from their academic programme. Furthermore, in order to confront the social stigma surrounding mental health and illustrate the widespread occurrence of these conditions we should be exposed to roleplays and case studies to understand mental illness and stigma and what we can do to assist the capacity of individuals to function normally, and provide recommendations for effective coping strategies.” (Participant 10)

Similarly, a student said:

“Mental health awareness is crucial because…Yes, education such as mental health campaigns or initiatives…Support for the acceptance of mental illnesses may increase the likelihood that individuals who are sick will seek help for their mental health problems, and thereby decreasing their sense of isolation, status loss, right?” (Participant 16).

## 4 Discussion

This study reflects students’ beliefs and attitudes toward patients who are experiencing mental illness and makes a valuable contribution to the existing literature, particularly in the United Arab Emirates. This study revealed that participants exhibited a basic understanding of mental illness, as evidenced by their ability to recognize a range of treatments and etiological factors associated with this condition. Although students had a basic understanding of mental illness and its treatment, stigmatizing attitudes were still present. One plausible explanation is that health science students’ attitudes may have been shaped by observing societal responses to mental illnesses, including media portrayals and community attitudes.

Despite participants’ understanding of common mental health issues such as anxiety and stress, a considerable proportion indicated a lack of practical expertise in overseeing patients with severe mental illnesses. Consequently, they were unprepared to assist patients with mental illnesses. Students’ perceived lack of preparedness and low confidence levels may hinder their willingness to support individuals with mental illnesses adequately.

The themes identified in this study, including perception and knowledge, unpacking stigma surrounding mental illness, attitudes toward mental illness, and advocacy and education, closely align with Corrigan and Watson's (2002) three constructs of stigma: public stigma, self‐stigma, and label avoidance. From the study findings, it was clear in the participants’ narratives that public stigma, or societal endorsement of negative stereotypes, was evident in students associating mental illness with violence and rejection. Self‐stigma emerged in participants’ concealment of mental illness to avoid social or academic repercussions. Label avoidance was reflected in delayed help‐seeking due to cultural and familial pressures (Corrigan and Watson 2002).

Specific individuals in the group described the challenges they faced when a family member was living with mental illness, which encompassed the strain and negative consequences of familial relationships. This finding aligns with prior investigations that recorded similar psychological burdens and sources of stress encountered by individuals providing support and attention to family members living with mental illness (Ntsayagae et al. 2019).

Positive changes in attitudes were noted when students interacted directly with individuals experiencing mental illnesses, leading to greater empathy and understanding. By engaging directly with patients, students could challenge pre‐existing stereotypes and develop more empathetic attitudes, thereby reducing stigma. These changes in prevailing attitudes are a testament to the personal factors, behaviors, and environmental influences that interact to shape attitudes (Buige et al. 2021). These factors directly influence resource allocation, treatment, communication, and the overall quality of care provided.

Numerous studies corroborate the finding that individuals with mental illnesses and their families often lack knowledge of the available treatment options (Rüsch et al. 2005). Healthcare workers are often assumed to be unaffected by the challenges faced by individuals with mental illness (Saridi et al. 2017). However, stigmatizing views persist even among educated health professionals (Picco et al. 2019; Subu et al. 2024). Our findings pointed out that individuals with severe mental illness are viewed less favorably than those with physical conditions. In another study, the participants demonstrated a basic understanding of mental illness, including its causes and treatments (Subu et al. 2024). However, some held negative attitudes, reflecting the widespread stigma observed among healthcare students in Indonesian universities (Subu et al. 2024).

Additionally, societal stigma surrounding mental illness was exacerbated by cultural perceptions, particularly in academic environments where students feared that mental health challenges could hinder their academic success. The need to foster a conducive campus environment that prioritizes self‐care and reduces the stigma surrounding mental illness was highlighted by participants as a critical factor in lessening the social stigma surrounding mental illness (Desai and Chavada 2018).

Many students demonstrated the ability to identify and understand primary concepts related to mental illness (Riffel and Chen 2020). According to the accounts of student participants, the health science program failed to provide an adequate mental illness education, which impeded their ability to provide suitable support to individuals with mental illnesses. Thus, students often lacked empathy toward those with mental health issues because of insufficient education in health science programs. Research has shown that mental health training can improve students’ ability to support those affected by mental illness by integrating supplementary subjects pertaining to mental illness into the academic curriculum and organizing periodic seminars. Social media content that advocates mental health is an additional effective intervention that should be considered (Fakhrunnisak and Patria 2022).

An increasing number of academic institutions are placing greater emphasis on the advancement of students’ mental well‐being (Gyaltshen et al. 2022, Clinton et al. 2025). The stigma surrounding mental illness on college campuses is largely attributed to two factors: a lack of understanding of mental illness and immense pressure to succeed academically. Students often hesitate to slow down or seek help because of the fear of jeopardizing their opportunities to succeed academically compared to their peers (Harris et al. 2018). In our study, the third‐year students expressed a mix of empathy and limited formal understanding regarding mental illness. This finding aligns with previous research indicating that while health science students may hold generally positive intentions, they often lack the depth of knowledge required to manage mental illness (Vidourek and Burbage 2019). Furthermore, Corrigan and Bink (2015) explain that stigma often continues when there is little education or personal contact, especially if mental illness is seen mainly as a medical problem (Corrigan and Bink 2015).

Many students were aware of both positive and negative societal perceptions of mental illness, and some expressed the negative impact of these attitudes on their health and social connections. Students recommended strategies such as education campaigns, training, and better access to resources to reduce stigma. Educational events showed the greatest impact (Chow et al. 2020). Using Corrigan's framework, stigma can be reduced through targeted education that promotes understanding and help‐seeking. Evidence‐based interventions, including campus awareness campaigns, to improve attitudes and mental health outcomes. Positive role models and empathetic healthcare professionals further reinforce stigma reduction. Universities can leverage online resources and align mental health initiatives with global best practices (Clinton et al. 2025).

### 4.1 Recommendations

First, mental illness instructions should be introduced earlier in the curriculum across all programs. Opportunities for student interactions with patients or simulations can strengthen mental health education and reduce stigma. Additionally, promoting observational learning through positive mental health role models will improve attitudes. Providing practical experience and simulated activities to enhance self‐efficacy can build students’ confidence in managing mental health issues. Further, it is recommended that universities adopt Corrigan's stigma reduction strategies—particularly education and contact‐based initiatives—through structured, evidence‐based events (Rüsch et al. 2005). These strategies should be supported by online resources and benchmarking practices to ensure alignment with global standards and sustained impact on student mental health outcomes(Clinton et al. 2025).

Creating a supportive campus environment that prioritizes mental health, along with awareness campaigns, can further reduce stigma. The cultural context in the UAE must be factored into the educational program. Reducing academic pressure and promoting a balance between academic success and mental well‐being are essential for students’ well‐being.

### 4.2 Study Limitations

This research relied on self‐reported data from a small sample. Additionally, because mental illness is a sensitive topic, participants may have provided responses they felt were socially acceptable instead of expressing their true beliefs and attitudes. For this study, only a limited number of participants from each health program were selected. As a result, differences among the healthcare programs were not considered. The small sample size and focus on a single university may impact the generalizability of our findings. The study's results may not be applicable in other contexts due to the small sample size, similar characteristics of participants, and the emphasis on one specific university. In other words, the limited scope of the qualitative design restricts the generalizability of these findings regarding their representation of healthcare student populations. Future research should investigate interdisciplinary differences and validate these results in the context of the United Arab Emirates.

## 5 Conclusion

This study highlights stigma in health science programs, with some students holding stigmatizing views. Therefore, early mental health education and anti‐stigma campaigns are urgently required. Collaboration between healthcare systems and educational institutions is essential. Additionally, further investigation into the stigma experienced by health science students toward individuals with mental illness is recommended.

## Conflicts of Interest

The authors declare no conflicts of interest.

## Data Availability

The data that support the findings of this study are available on request from the corresponding author. The data are not publicly available due to privacy or ethical restrictions.
